# Multimedia sister circles: a study protocol advancing an intergenerational approach to sexual health and HIV prevention for black women and girls (RoyalTea study)

**DOI:** 10.3389/fpubh.2026.1799714

**Published:** 2026-05-28

**Authors:** Megan T. Ebor, Bhjan Kaur, Madeline Y. Sutton

**Affiliations:** 1 School of Social Work, College of Health and Human Services, San Diego State Univeristy, San Diego, CA, United States; 2 Morehouse School of Medicine, Atlanta, GA, United States

**Keywords:** Black women, HIV-human immunodeficiency virus, inter-generational, mixed-methods, prevention, sexual health, multimedia, study protocol

## Abstract

**Background:**

Black women in the United States experience disproportionately high rates of Human Immunodeficiency Virus (HIV) and other sexually transmitted infections (STIs), shaped by social determinants, stigma, and limited access to culturally responsive sexual health interventions. Health communication film, community-based, and intergenerational approaches have shown promise in promoting engagement and dialogue around sensitive health topics, yet brief, scalable interventions tailored to Black women across the life course remain underexplored.

**Methods:**

This protocol describes a mixed-methods, pre–post, single-session intergenerational group intervention designed to evaluate the feasibility and potential impact of *RoyalTea: Mind, Body, and Soul Care*, a film-centered Sister Circle intervention. Approximately 200 Black women aged 18 years and older, living with or vulnerable to HIV, will be recruited from community-based settings using purposive strategies. Each intervention session lasts approximately 2.5–3 h and includes HIV and sexual health education, viewing of a 25-min health communication film (*Even Me*), pre- and post-intervention surveys, and a facilitated intergenerational Sister Circle discussion. Quantitative measures assess HIV-related motivation and behavioral intentions, HIV prevention self-efficacy, and HIV knowledge before and after the intervention. Qualitative data are collected through open-ended post-survey items and semi-structured group discussions to examine participants' interpretations of sexual health, HIV prevention, stigma, and community responsibility.

**Analysis:**

Pre–post change in quantitative outcomes will be examined using paired statistical analyses. Qualitative data will be analyzed thematically to explore perceived impact, meaning-making, and mechanisms of change. Quantitative and qualitative findings will be integrated during interpretation to provide a comprehensive assessment of intervention feasibility and potential effects.

**Discussion:**

This study will generate evidence on the feasibility of a brief, intergenerational, multimedia-based Sister Circle intervention for promoting sexual health and HIV prevention among Black women. Findings will inform future intervention refinement and larger-scale evaluations, and contribute to the development of culturally grounded, community-engaged strategies to address HIV-related inequities across the life course for Black women.

## Introduction

Sexual health and wellness remain critical yet underaddressed components of overall health, with persistent disparities affecting Black women across the life course. In the United States, Black women continue to experience disproportionately high rates of Human Immunodeficiency Virus (HIV) and other sexually transmitted infections (STIs). According to the Centers for Disease Control and Prevention (CDC), HIV diagnosis rates among Black females remain the highest among women nationally. In 2023, rates among Black women were approximately three times higher than those among Hispanic or Latine women and eleven times higher than those among White females. Black women also accounted for approximately half of new HIV diagnoses among women, despite representing a substantially smaller proportion of the female population (1).

These disparities reflect enduring inequities in access to prevention resources, affirming healthcare, and social conditions that shape exposure and engagement across the HIV prevention and care continuum. Structural racism, gender inequity, and adverse social determinants of health, including housing instability, economic insecurity, and limited access to transportation and culturally responsive services, continue to constrain opportunities for consistent prevention and care (2–5). Stigma, discrimination, and poverty further operate as barriers across prevention and treatment pathways, reinforcing the need for interventions that extend beyond individual-level behavior change to address interpersonal, social, and structural contexts (2, 6–9).

Research demonstrates that intersecting social identities, including race, gender, sexuality, and age, shape how Black women experience stigma and discrimination in sexual health settings (10–13). Intersectional stigma has been associated with reduced engagement in health services and poorer sexual health outcomes, particularly when compounded by medical mistrust rooted in historical and contemporary harms (11, 12, 14–16). These findings underscore the importance of culturally grounded approaches that prioritize trust-building, relational safety, and community strengths. Narrative health communication strategies (using storytelling to make health information engaging) that center lived experience and authenticity have shown promise in increasing engagement, improving relevance of health information, and supporting sustained dialogue around stigmatized health topics (17–19).

To address these gaps, we developed *RoyalTea: Mind, Body, and Soul Care*, a brief, single-session, multimedia Sister Circle intervention that integrates education, narrative film, and facilitated group dialogue. Sister Circles are culturally congruent, peer-based group formats that have been widely used among Black women to promote health education, social support, and collective empowerment. Foundational scholarship describes Sister Circles as relational spaces that leverage shared storytelling, mutual affirmation, and collective meaning-making to support wellbeing (20). More recent studies demonstrate the feasibility and acceptability of Sister Circle-based interventions across a range of contexts, including mental health, stress, and coping (21–23). Evidence also supports the relevance of Sister Circle models among middle-aged and older Black women, including improvements in psychosocial outcomes and sustained engagement (24, 25). And although not reported in scientific literature, Dr. Maya Angelou beautifully describes the part of Black women's relationships that is often unmeasured as “those who provide profound support, stand for you when you cannot, and with whom you share laughter, secrets, and life (26).” The goal of *RoyalTea* was to tap into all of that.

Another defining feature of the RoyalTea intervention is its intentional intergenerational design. The intervention convenes Black women across adulthood, beginning at age 18, to support reciprocal learning and life-course-informed dialogue. Intergenerational interventions have been shown to facilitate bidirectional knowledge exchange, strengthen social cohesion, and promote protective health behaviors by drawing on the complementary perspectives of different generations (27–32). In sexual health contexts, intergenerational engagement may help disrupt silence, normalize ongoing conversations across the life course, and foster collective accountability.

RoyalTea is also distinguished by its central use of health communication film. The intervention is anchored by *Even Me*, a health communication film designed to challenge myths related to sexual health, aging, intimacy, and HIV while centering lived experiences of Black women, a Latina, and one man across the life course (17–19, 33). Rather than functioning as a supplemental educational tool, the film serves as the primary catalyst for reflection and dialogue. Integrating shared film viewing into a culturally grounded and intergenerational Sister Circle format supports psychologically safer discussion of sensitive topics, including sexual health, HIV prevention, testing intentions, and stigma-related experiences.

### Purpose and aims

The purpose of this protocol is to evaluate the feasibility and potential impact of a single-session (2.5–3 h) film-based Sister Circle intervention, as part of the RoyalTea: Mind, Body, and Soul care initiative, on HIV prevention-related outcomes among approximately 200 Black women living with or vulnerable to HIV. Consistent with the study design and measures, the evaluation focuses on pre–post changes in HIV-related motivation and behavioral intentions, HIV prevention self-efficacy, and HIV knowledge. The study also examines how participants interpret and make meaning of HIV-related stigma, strength, and community responsibility through qualitative data.

We hypothesize that participation in the RoyalTea intervention will be associated with increased HIV knowledge, improved HIV prevention self-efficacy, and stronger motivation and intentions related to HIV testing, prevention communication, and stigma-challenging behaviors.

The specific aims are:

Aim 1. To use mixed methods to evaluate pre-post change in HIV prevention-related outcomes following participation in the RoyalTea single-session intervention. We will examine HIV-related motivation and behavioral intentions, HIV prevention self-efficacy, and HIV knowledge among approximately 200 Black women.

Aim 2. To examine how participants experience and interpret HIV prevention, sexual health promotion, and HIV-related stigma and strength narratives following the film-centered Sister Circle session, using qualitative data from post-film open-ended survey items and semi-structured intergenerational group discussions.

## Methods

### Study design

This study employs a mixed-methods, pre-post intergenerational group intervention design to evaluate the impact of a film-centered Sister Circle intervention on sexual health-related outcomes among Black women living with or vulnerable to HIV. The intervention utilizes an intergenerational Sister Circle model that brings together Black women from multiple age cohorts within shared group sessions to promote reciprocal learning, collective reflection, and life-course-informed dialogue around sexual health, HIV prevention, and wellness.

The study is grounded in evidence demonstrating that culturally grounded, narrative-based, and intergenerational interventions are effective in improving health knowledge, self-efficacy, and stigma-related outcomes. Accordingly, this study hypothesizes that participation in the RoyalTea intervention is associated with increased sexual health self-efficacy, enhanced HIV knowledge and awareness, and reductions in HIV-related stigma (17, 18, 27). It is further hypothesized that the intergenerational Sister Circle format will be perceived as acceptable, culturally resonant, and impactful, and that specific narrative and relational elements of the film-centered discussions will contribute meaningfully to observed change in outcomes (knowledge, motivations, and attitudes).

### Mixed-methods analytic approach

This study employs a convergent mixed-methods analytic approach, in which quantitative and qualitative data are collected within the same intervention session, analyzed separately, and integrated during interpretation to provide complementary insights into intervention feasibility, potential impact, and mechanisms of change. This approach is appropriate for evaluating complex, community-based interventions that target behavioral, psychosocial, and contextual outcomes and aligns with mixed-methods reporting guidance for public health intervention research.

### Study setting, sample, and recruitment

The study sample consists of approximately 200 Black women recruited from a range of national community-based settings, such as HIV service agencies and entities that serve Black women. Recruitment utilizes purposive, community-centered strategies designed to leverage existing social networks and trust relationships. These strategies include snowball sampling, word-of-mouth referrals, (with many seeds being students at San Diego State University and participants from local community-based organizations that outreach to women) and outreach via social media platforms (Instagram). Such approaches are intended to engage women who may be less likely to participate in traditional research settings (34).

Group-based film screenings and intergenerational Sister Circle sessions include 10–20 participants per group, a size selected to facilitate meaningful dialogue, peer connection, and collective learning while maintaining psychological safety (35). The multimedia (including a power point presentation, film, zoom, and audio recordings) intervention sessions are delivered both in person and virtually via Zoom to maximize accessibility and reduce participation barriers related to transportation, scheduling, and geographic location.

### Inclusion criteria

Participants are eligible for inclusion if they:

- Self-identify as Black
- Self-identify as a cisgender or transgender woman
- Are 18 years of age or older
- Are able to participate in English
- Are able to participate either in person or virtually via Zoom

Participants report their city and state of residence to support contextual characterization of the sample. HIV status and HIV vulnerability are assessed via self-report within the pre-intervention questionnaire.

### Exclusion criteria

Individuals are excluded from participation if they are unable to provide informed consent. In such cases, participants are provided with referrals to appropriate services as needed.

### Study procedures

All study activities are conducted in accordance with a standardized protocol designed to ensure consistency and replicability across intervention sessions. Consistent with community-engaged (including community members in various aspects of study creation, delivery, and feedback) research principles that emphasize trust-building, cultural congruence, and shared understanding as central to ethical research practice and meaningful participant engagement, intervention facilitators are intentionally racially and culturally aligned with participants (36). This approach is designed to promote relational safety, reduce power differentials, and support open dialogue around sensitive topics such as sexual health and HIV.

The study protocol was reviewed and approved by the San Diego State University Institutional Review Board (IRB #IRB-24-0267). Participants are screened for eligibility prior to enrollment and, once eligibility is confirmed, are assigned to an intervention session that aligns with their availability, scheduling preferences, and location to support participation and retention.

Each RoyalTea intervention session lasts approximately 2.5–3 h and follows a structured sequence of activities implemented consistently across both in-person and virtual formats (Table 1). Sessions are facilitated by trained members of the research team with experience in Sister Circle facilitation and culturally responsive group engagement.

**Table 1:** RoyalTea intervention procedures (single-session delivery).

Session component	Domains covered	Timing
Welcome, informed consent, and icebreaker	Facilitated welcome; review of study purposes, procedures, risks, and benefits; informed consent obtained; icebreaker to establish group norms, rapport, and psychological safety.	30 min
Pre–intervention surveys	Completion of baseline (pre–film) surveys assessing HIV–related motivation, behavioral intentions, self–efficacy, knowledge, and demographics.	30 min
HIV and sexual health education (HIV 101)	Brief, culturally affirming overview of HIV transmission, prevention, testing, stigma, and wellness across the life course, including current prevention and treatment advancements.	25 min
Film screening (Even Me)	Group viewing of a 25–min health communication film serving as the central narrative stimulus for reflection and dialogue.	25 min
Intergenerational Sister Circle discussion	Semi–structured, facilitated group discussion guided by film content and standardized prompts, emphasizing shared storytelling, intergenerational dialogue, stigma and strength narratives, and intended actions.	40 min
Post–intervention surveys	Completion of post–intervention (post–film) surveys assessing HIV–related motivation, behavioral intentions, self–efficacy, knowledge, and demographics.	30 min
Session close and incentive distribution	Closing reflections, participant questions, and distribution of participant incentive upon completion of all session components.	10 min

All intervention components are delivered within a single session lasting approximately 2.5–3 h and are implemented consistently across both in–person and virtual formats.

### Intervention procedures (single-session delivery)

#### Step 1: Welcome, informed consent, and icebreaker

Sessions begin with a welcome, review of study procedures, and an informed consent process lasting approximately 30 min. Facilitators explain the study purpose, procedures, risks, and benefits; respond to participant questions; and obtain written informed consent. A facilitated icebreaker follows, designed to establish rapport, introduce group norms, and foster a supportive and respectful environment conducive to open dialogue.

#### Step 2: Pre-intervention surveys

Immediately before the session, participants complete pre-intervention questionnaires administered electronically via Qualtrics accessed through secure email links. Each survey requires approximately 30 min to complete and includes validated measures assessing HIV knowledge, HIV self-efficacy, HIV-related stigma, and self-reported HIV status. Participation in the surveys is voluntary.

#### Step 3: HIV education

Following informed consent, participants engage in a facilitated HIV education segment lasting approximately 25 min. The education component included an HIV foundational overview designed to establish a shared baseline of knowledge across participants. Content addressed core concepts related to HIV transmission, prevention strategies, testing, treatment, and contemporary prevention advancements, including long-acting injectable modalities. The session emphasized sexual health and wellness across the life course and was delivered in a culturally affirming, nonjudgmental manner to support informed participation in subsequent discussion.

#### Step 4: Film screening

Participants then view the first 25 min of *Even Me the Journey*, a health communication film, in a group setting. The film serves as the central narrative stimulus for the intervention and is designed to challenge myths related to sexual health, aging, intimacy, and HIV while centering the lived experiences of Black women and one man. *Even Me the Journey* is publicly available via Fawesome (https://fawesome.tv/movies/10698896/even-me-the-journey), supporting transparency and potential replication of the intervention.

#### Step 5: Post-intervention surveys

Immediately after the session, participants complete the same questionnaires post-intervention. To enable paired analyses while maintaining confidentiality, pre- and post-surveys are linked using a numerical identifier assigned to each participant; no identifying information is included in the survey data.

#### Step 6: Intergenerational sister circle discussion

Following completion of the post-test questionnaire, participants engage in a semi-structured, facilitated Sister Circle discussion lasting approximately 40 min. Guided by the film content, facilitators prompt dialogue related to HIV prevention, safer sex practices, HIV-related stigma, sexual health, and overall wellness. The Sister Circle format emphasizes shared storytelling, intergenerational exchange, and collective meaning-making. Facilitators record structured field notes and audio recordings of the discussions, which are transcribed verbatim for analysis; transcripts and notes do not include identifying information.

### Participant incentives

Participants who complete the full intervention session, including surveys and Sister Circle discussion, receive a $60 incentive in recognition of their time and contribution to the study.

## Assessment time and instruments

### Quantitative and qualitative assessment instruments

#### Quantitative survey instruments

Even Me RoyalTea Film Questionnaire (Motivation and Behavioral Intention) HIV-related motivation and behavioral intentions are assessed using a 10-item Even Me RoyalTea film questionnaire developed for this intervention and administered at baseline (T0, pre-sister circle) and post-intervention (T1, post-sister circle). The measure captures two domains.

The motivation domain (Items 1–5) assesses motivation to learn about HIV prevention, interest in HIV testing, willingness to discuss HIV testing with others, perceived personal responsibility to reduce HIV-related stigma, and beliefs regarding the importance of open dialogue for community health.

The behavioral change intention domain (Items 6–10) assesses intentions to obtain HIV testing, initiate conversations about testing, share HIV prevention information, challenge stigma in social contexts, and seek additional HIV-related educational resources.

Items are rated on 5-point Likert scales assessing agreement or likelihood. At T1, items are phrased to assess perceived change following the film viewing and Sister Circle experience.

**HIV Self-Efficacy Scale** HIV prevention self-efficacy is assessed using the 9-item HIV Self-Efficacy Scale developed by Smith et al. (40) and administered at T0 and T1. The scale measures confidence in performing HIV risk-reduction behaviors, including condom negotiation, refusal of unwanted or unsafe sex, communication with sexual partners, and refusal of injection-related risk behaviors. Items are rated using a Likert-type confidence scale, with higher scores indicating greater self-efficacy.

**HIV Knowledge Questionnaire** HIV knowledge is assessed using an 11-item HIV Knowledge Questionnaire adapted from Heckman et al. (41) and administered at T0 and T1. Items assess understanding of HIV transmission, condom use, lubrication, and common misconceptions, such as asymptomatic infection and withdrawal practices. Response options include *True, False, Don't know*, or *Refuse*.

### Post-intervention open-ended survey items

At T1, participants complete three open-ended survey items designed to capture immediate reflections on the film and Sister Circle experience. Items explore (1) the most impactful message or moment from the film, (2) perceived changes in perspectives related to HIV testing, prevention, or stigma, and (3) actions participants intend to take following the intervention. These responses provide qualitative context for observed quantitative changes and support mixed-methods integration.

### Semi-structured post-film sister circle discussion

Following completion of the post-intervention survey, participants engage in a semi-structured, facilitated Sister Circle discussion lasting approximately 40 min. The discussion guide includes 19 open-ended prompts (derived from literature reviews and recent reports) organized across thematic domains, including emotional reactions to the film; learning related to HIV and aging; stigma and strength narratives; intersectional influences of race, age, and gender; behavioral intentions; and collective and individual responsibility for sexual health and wellness.

Discussions are audio-recorded with participant consent and supplemented by structured facilitator field notes. Audio recordings are transcribed verbatim and de-identified prior to analysis. Discussion data are used to examine how participants interpret HIV prevention, stigma, resilience, and community responsibility following the intervention and to contextualize pre–post survey findings (Table 2).

**Table 2:** Measures and assessment timepoints for the royaltea sister circle intervention.

Assessment tool	Domains covered	Number of items	Response format	Assessment timepoints
RoyalTea Film Questionnaire (Even Me)	HIV–related motivation; HIV prevention behavioral intentions; stigma–related responsibility	10	5–point Likert scales (agreement and likelihood)	Pre–film and post–film
HIV Self–Efficacy Scale (40)	HIV prevention self–efficacy (condom use, partner communication, refusal of risky behaviors)	9	Likert–type confidence scale	Pre–film and post–film
HIV Knowledge Questionnaire (41)	HIV transmission, condom use, lubrication, misconceptions	12	True / False / Don't know / Refuse	Pre–film and post–film
Demographic and health characteristics questionnaire	Sociodemographic characteristics, sexual behavior, health status, healthcare utilization	12	Categorical and self–report items	Pre–film only
Post–Film Open–ended survey items	Perceived impact, changes in perspectives, intended actions	3	Open–ended text responses	Post–film only
Semi–Structured Sister Circle discussion guide	Film reactions; HIV learning; stigma and strength narratives; behavioral intentions; community action	19 guiding questions	Facilitated group discussion with audio recording and field notes	Post–film only

Pre- and post-film measures are linked using numeric identifiers to allow paired analyses while maintaining participant confidentiality. Stigma is examined through motivation- and intention-related items and qualitative narrative data rather than a validated stigma scale.

### Data management and confidentiality

All study data are managed in accordance with approved Institutional Review Board protocols and established data security procedures. Survey data are collected electronically using Qualtrics for participants completing assessments online. For in-person sessions, participants are offered the option to complete surveys either electronically via Qualtrics or in paper-and-pencil format to accommodate individual preferences and accessibility needs. Privacy for participants was assured to decrease the possibility of mode effect (a systematic difference that is attributable to the mode of data collection).

For participants who complete paper surveys, responses are entered into the Qualtrics database by two trained members of the research team immediately following the session. Each team member independently reviews the entered data to conduct quality control checks and ensure accuracy and completeness. Any discrepancies identified during this process are resolved through review of the original paper surveys.

All survey data are linked using numeric identifiers rather than personal identifiers to maintain confidentiality and enable paired pre–post analyses. Audio recordings from Sister Circle discussions are securely stored and transcribed verbatim, with all identifying information removed during transcription. Paper surveys and consent forms are stored in locked filing cabinets accessible only to authorized research personnel, and electronic data are stored on password-protected, encrypted servers. Access to identifiable data is limited to approved members of the research team.

## Anticipated results

It is anticipated that participation in the RoyalTea film-centered Sister Circle intervention will be associated with positive pre–post changes across key HIV prevention–related outcomes. Specifically, we expect participants to demonstrate increased HIV-related motivation and behavioral intentions following the intervention, including greater interest in HIV testing, increased willingness to discuss HIV testing and prevention with others, and stronger intentions to engage in stigma-challenging behaviors within their social networks.

We further anticipate improvements in HIV prevention self-efficacy, as reflected in increased confidence in condom negotiation, refusal of unwanted or unsafe sexual encounters, and communication with sexual partners. These changes are expected to be observed immediately following the intervention, consistent with the single-session design and the use of pre–post measures administered within the same session.

Participants are also expected to demonstrate changes in HIV knowledge, particularly with respect to common misconceptions related to HIV transmission, condom use, lubrication, and asymptomatic infection. Given the combination of brief education and narrative film exposure, we anticipate that knowledge changes will be evident across multiple items on the HIV Knowledge Questionnaire.

Although the study does not include a validated HIV stigma scale, we anticipate shifts in stigma-relevant attitudes and intentions as reflected in the qualitative data. Quantitatively, participants are expected to report increased perceived responsibility to address HIV-related stigma and greater intention to challenge stigmatizing beliefs or behaviors. Qualitatively, post-intervention open-ended responses and Sister Circle discussions are expected to reveal nuanced reflections on stigma, strength, resilience, and collective responsibility, as well as increased openness to discussing HIV and sexual health across generations.

Qualitative analysis of the semi-structured Sister Circle discussions is anticipated to yield themes related to emotional resonance with the film, increased awareness of HIV risk and prevention across the life course, and the perceived value of intergenerational dialogue in disrupting silence around sexual health and HIV. Participants are expected to articulate how the film and group discussion facilitated shared meaning-making, normalized conversations about HIV testing and prevention, and supported feelings of connection, validation, and empowerment.

Across both quantitative and qualitative data, we anticipate high levels of intervention acceptability and perceived relevance. Participants are expected to report that the film-centered Sister Circle format was culturally resonant, emotionally engaging, and appropriate for addressing sensitive topics. The intergenerational design is anticipated to be viewed as a strength, facilitating reciprocal learning and fostering empathy and understanding across age groups.

Finally, we anticipate that findings from this study will demonstrate the feasibility of implementing a brief, single-session, multimedia Sister Circle intervention in both in-person and virtual formats. Collectively, anticipated results are expected to inform future refinement of the RoyalTea intervention and provide preliminary evidence to support larger-scale evaluations examining sustained outcomes over time.

## Baseline assessments

### Demographic and health characteristics

At baseline (T0), participants complete a demographic and health characteristics questionnaire assessing age, gender identity, sexual orientation, education, household income, employment status, relationship status, household composition, self-rated health, health insurance status, sexual activity in the past 12 months, HIV status (if they choose to disclose), and timing of most recent healthcare visit. These variables are used to describe the sample and to contextualize quantitative and qualitative findings.

## Statistical analyses

### Sample size and power calculation

Power calculations were conducted as sensitivity analyses for within-person pre–post change. With N=200 participants and a two-sided α = 0.05, the study has 80% power to detect a small standardized mean change (Cohen's d_z≈0.20) and 90% power to detect d_z≈0.23. Sensitivity analyses incorporating potential group clustering (average m = 15; ICC = 0.01–0.05) indicate detectable effects ranging from d_z≈0.21 to 0.26.

### Data analysis

#### Quantitative analysis

Quantitative analyses will assess pre–post changes in HIV prevention–related outcomes following participation in the RoyalTea intervention. Descriptive statistics will be used to summarize baseline demographic, health, and sexual behavior characteristics of the sample.

Primary quantitative outcomes include:

1. HIV-related motivation and behavioral intentions (RoyalTea Film Questionnaire),
2. HIV prevention self-efficacy (HIV Self-Efficacy Scale),
3. HIV knowledge (HIV Knowledge Questionnaire).

For each outcome, composite scores will be calculated where appropriate. Internal consistency will be examined descriptively for multi-item measures. Pre–post changes from baseline (T0) to post-intervention (T1) will be evaluated using paired statistical tests appropriate to the distribution of the data (e.g., paired *t* tests or nonparametric equivalents). Effect sizes (Cohen's *d* for paired designs) and 95% confidence intervals will be reported to quantify the magnitude of observed change.

Given the group-based delivery of the intervention, sensitivity analyses will examine the potential influence of clustering at the Sister Circle session level. Where appropriate, mixed-effects models with time as a fixed effect and random intercepts for participants and/or session groups will be explored to assess the robustness of findings. Statistical significance will be set at *p* < 0.05 for primary analyses, with interpretation emphasizing effect sizes and practical significance.

Analyses will be restricted to participants with matched pre–post survey data linked via numeric identifiers. Patterns of missing data will be examined and reported. Quantitative analyses will be conducted using standard statistical software (e.g., SPSS, R, or Stata).

### Qualitative analysis

Qualitative data will be derived from two sources: (1) post-intervention open-ended survey responses and (2) audio-recorded, semi-structured Sister Circle discussions. Audio recordings will be transcribed verbatim and de-identified prior to analysis. Facilitator field notes will be used to supplement transcript data and provide contextual detail.

Qualitative analysis will follow a thematic analysis approach. An initial codebook will be developed using a combination of deductive codes informed by the study aims (e.g., HIV prevention learning, testing intentions, stigma and strength narratives, intergenerational dialogue, community responsibility) and inductive codes emerging from the data. Two trained analysts will independently code a subset of transcripts to establish coding consistency and refine the codebook. Discrepancies will be resolved through discussion and consensus.

Themes will be identified through iterative comparison within and across data sources, with attention to patterns related to meaning-making, emotional resonance with the film, perceived relevance across the life course, and intentions for individual and collective action. Analytic rigor will be supported through reflexive memo writing, team debriefs, and documentation of analytic decisions. Qualitative data management and coding will be supported using qualitative analysis software (NVivo).

### Integration of quantitative and qualitative data

**Figure 1:**
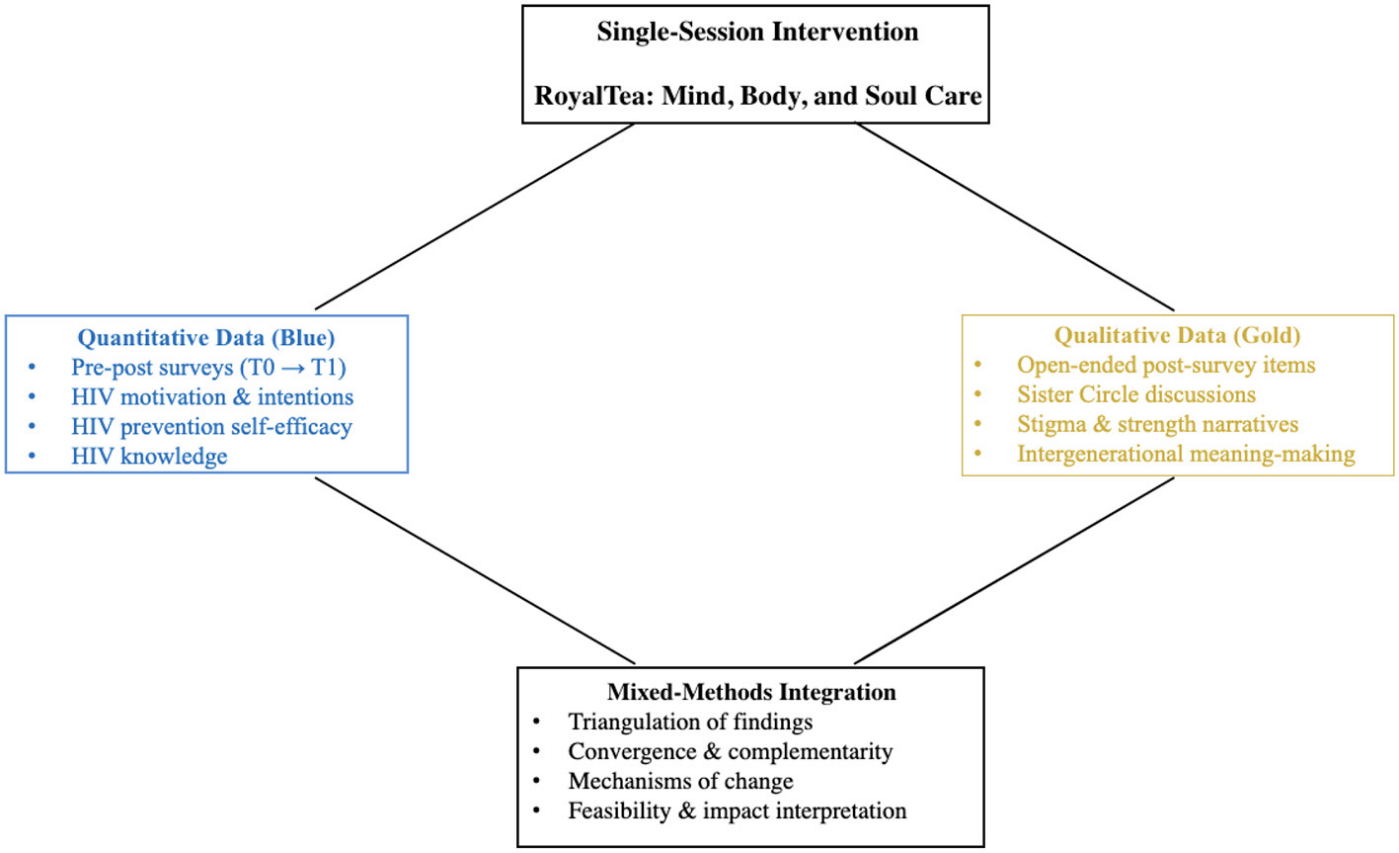
Convergent mixed-methods integration for the RoyalTea study. Quantitative and qualitative data are collected within a single-session intervention, analyzed in parallel, and integrated during interpretation to examine feasibility, short-term outcomes, and mechanisms of change related to HIV prevention and sexual health promotion.

Integration will occur during the interpretation phase using a convergent design, consistent with mixed-methods best practices (Figure 1). Quantitative results will be compared with qualitative themes to examine convergence, complementarity, and divergence across data sources. Qualitative findings will be used to contextualize and explain observed quantitative changes, particularly with respect to motivation, self-efficacy, knowledge changes, and stigma-related intentions.

This integrative approach will allow the study to assess not only whether change is observed following the intervention, but also how participants interpret and experience that change within their social, cultural, and intergenerational contexts. Integration will support a more nuanced understanding of intervention mechanisms and inform future refinement and scale-up.

### Analytic considerations for protocol reporting

Given the single-session design and immediate post-intervention assessment, analyses will focus on short-term changes and participant-reported experiences rather than sustained behavioral outcomes. Findings will be interpreted as preliminary and hypothesis-generating, consistent with protocol objectives, and will inform future longitudinal evaluations and controlled trials.

## Discussion

This protocol describes the design and analytic approach for evaluating *RoyalTea: Mind, Body, and Soul Care*, a brief, film-centered, intergenerational Sister Circle intervention aimed at promoting sexual health, HIV prevention, and stigma-related dialogue among Black women across the life course.

### Centering personhood in sexual health and HIV prevention

A key contribution of the RoyalTea protocol is its explicit centering of personhood in sexual health and HIV prevention. Rather than framing HIV prevention solely in terms of individual risk behaviors, the intervention foregrounds participants' identities, lived experiences, relationships, and values. The use of narrative film and facilitated Sister Circle dialogue creates space for participants to articulate how sexual health, prevention, and stigma intersect with their own life histories and social contexts. Quantitative measures assessing motivation, behavioral intentions, and self-efficacy are complemented by qualitative data that capture meaning-making, emotional resonance, and perceived responsibility, reflecting a holistic understanding of sexual health that extends beyond knowledge acquisition alone.

This person-centered orientation aligns with calls to move away from deficit-based models of HIV prevention toward approaches that affirm agency, dignity, and self-determination (37). By prioritizing how participants interpret and internalize prevention messages, the protocol advances a more nuanced and humanizing approach to sexual health promotion.

### Leveraging community strengths through sister circles and narrative engagement

The RoyalTea intervention is grounded in the recognition that community strengths are essential resources for advancing sexual health and wellness. Sister Circles function as culturally congruent spaces that leverage shared storytelling, mutual affirmation, and collective knowledge-building. Embedding the intervention within an intergenerational Sister Circle format allows participants to draw on communal wisdom and relational support, reinforcing prevention messages through social connection rather than individual instruction alone. One limitation is in trying to ensure intergenerational ages when recruiting for different Sister Circles; intentional assignment by ages across groups may be an important strategy to ensure range.

The central use of a health communication film further amplifies these strengths by providing a shared narrative reference point that facilitates dialogue across generations. Narrative engagement reduces barriers to discussing stigmatized topics and enables participants to engage with HIV prevention and sexual health in ways that feel accessible and culturally resonant (38). The mixed-methods design is well suited to examining how these community-driven processes shape engagement, acceptability, and perceived impact.

### Addressing social determinants of sexual health inequities

The protocol also attends to social determinants that shape sexual health and HIV-related outcomes. By offering the intervention in both in-person and virtual formats and allowing flexible scheduling, the study seeks to reduce structural barriers related to transportation, time constraints, and access. Recruitment strategies that leverage community networks reflect an understanding of how historical exclusion and medical mistrust influence participation in health research (39).

Although social determinants are not directly manipulated within the intervention, the study's design acknowledges their influence and positions RoyalTea as a complementary strategy within broader efforts to address systemic drivers of HIV disparities. Qualitative data from Sister Circle discussions are expected to illuminate how participants understand and navigate these structural conditions, providing insight into how individual and community-level interventions can be situated within larger systems of care and prevention.

### Measurement strategy and mixed-methods integration

The assessment strategy reflects a pragmatic balance between methodological rigor and feasibility for a single-session intervention. Quantitative outcomes focus on proximal indicators of change, including motivation, behavioral intentions, self-efficacy, and knowledge. Stigma is examined through motivation- and intention-related items and through rich qualitative narratives. This approach aligns with contemporary perspectives that conceptualize stigma as a socially embedded and context-dependent phenomenon rather than a static individual attribute. However, not assessing stigma using a quantitative measure is also a limitation. Although validated stigma scales exist, many do not fully capture the intersectional and context-specific experiences of stigma among Black women, including the ways racism, sexism, and HIV-related stigma are intertwined. As such, we prioritized qualitative approaches to allow for more nuanced, participant-driven insights. Future research should consider incorporating or developing quantitative measures that better reflect intersectional stigma and are responsive to change in intervention contexts.

The convergent mixed-methods design supports integration of quantitative and qualitative findings at the interpretation stage, enabling triangulation and deeper understanding of how narrative engagement, intergenerational dialogue, and community context contribute to observed changes. This integrative approach strengthens the protocol's relevance to public health practice and theory.

### Implications for public health practice and future research

If found feasible and acceptable, the RoyalTea model may offer a scalable, community-engaged approach to sexual health and HIV prevention that complements clinical and structural interventions. As a protocol, this study is designed to generate preliminary evidence and inform future research. Subsequent studies should examine longer-term outcomes, incorporate validated stigma measures, and evaluate integration with healthcare and prevention services. Additional work may also explore adaptation for other communities and settings.

### Conclusion

This protocol advances an integrated, equity-oriented approach to sexual health and HIV prevention by centering personhood, leveraging community strengths, and acknowledging social determinants that shape health outcomes. Through its mixed-methods design and culturally grounded intervention model, the RoyalTea study contributes to the growing body of public health research focused on developing resonant, accessible, and community-driven strategies to address sexual health disparities across the life course, especially for Black women.
